# Appreciation message

**DOI:** 10.1590/S2237-96222025v34e20250003.en

**Published:** 2025-09-08

The Editorial Core Group of Epidemiology and Health Services: Journal of the Brazilian National Health System (Epidemiologia e Serviços de Saúde: revista do SUS - RESS) extends its thanks to the ad hoc reviewers listed below for their collaboration and commitment to the careful assessment of manuscripts in 2024, which greatly contributed to the quality, independence and ethical rigor of the journal.

**Table:** 

Name	Institution	State	País
Adail Afrânio Marcelino Nascimento	Secretaria Municipal da Saude de Fortaleza	Ceará	Brazil
Adeânio Almeida Lima	Faculdade Estácio Alagoinhas	Bahia	Brazil
Adeylson Guimarães Ribeiro	Universidade de São Paulo	São Paulo	Brazil
Adinaura Gama de Castro	Santa Casa de Misericórdia do Pará	Pará	Brazil
Adriana Barni Truccolo	Universidade Estadual do Rio Grande do Sul	Rio Grande do Sul	Brazil
Adriana Cezaretto	Universidade Nove de Julho	São Paulo	Brazil
Adriana Lúcia Meireles	Universidade Federal de Ouro Preto	Minas Gerais	Brazil
Adriana Sanudo	Universidade Federal de São Paulo	São Paulo	Brazil
Adriana Silva	Universidade Federal da Bahia	Bahia	Brazil
Adriana Tavares de Moraes Atty	Instituto Nacional de Câncer	Rio de Janeiro	Brazil
Andréa Cronemberger Rufino	Universidade Estadual do Piauí	Piauí	Brazil
Alberto Madeiro	Universidade Estadual do Piauí	Piauí	Brazil
Alcindo José Rosa	Universidade Federal do Mato Grosso	Mato Grosso	Brazil
Alex Sandro Rolland Souza	Instituto de Medicina Integral Professor Fernando Figueira	Recife	Brazil
Alexandre Tadashi Inomata Bruce	Secretaria Municipal de Saúde de Manaus	Amazonas	Brazil
Aline do Nascimento	Ministério da Saúde	Distrito Federal	Brazil
Aline Fernanda Silva Sampaio	Universidade Federal do Acre	Acre	Brazil
Aline Pilon	Ministério da Saúde	Distrito Federal	Brazil
Aline Pinto Marques	Fundação Oswaldo Cruz	Rio de Janeiro	Brazil
Aline Sales	Universidade Federal de Minas Gerais	Minas Gerais	Brazil
Aline Vieira de Lima	Ministério da Saúde	Distrito Federal	Brazil
Alisson Diego Machado	Universidade de São Paulo	São Paulo	Brazil
Almária Mariz Batista	Universidade Federal do Rio Grande do Norte	Rio Grande do Norte	Brazil
Aluísio de Oliveira	Instituto Federal do Sudeste de Minas Gerais	Minas Gerais	Brazil
Amanda Cilene Cruz Aguiar Castilho da Silva	Universidade Federal do Rio de Janeiro	Rio de Janeiro	Brazil
Amanda Coutinho de Souza	Organização Pan-Americana da Saúde	Distrito Federal	Brazil
Amanda Fonseca Medeiros	Universidade Federal de Minas Gerais	Minas Gerais	Brazil
Amanda Nathale Soares	Escola de Saúde Pública do Estado de Minas Gerais	Minas Gerais	Brazil
Ana Carolina Bezerra de Lima	Universidade de Pernambuco	Pernambuco	Brazil
Ana Carolina Feldenheimer da Silva	Universidade do Estado do Rio de Janeiro	Rio de Janeiro	Brazil
Ana Carolina Micheletti Gomide Nogueira de Sá	Universidade Federal de Minas Gerais	Minas Gerais	Brazil
Ana Coelho de Albuquerque	Instituto de Medicina Integral Prof. Fernando Figueira	Pernambuco	Brazil
Ana Débora Assis Moura	Secretaria da Saúde do Estado do Ceará	Ceará	Brazil
Ana Freitas Ribeiro	Instituto de Infectologia Emílio Ribas	São Paulo	Brazil
Ana Isabelle de Gois Queiroz	Centro Universitário Uniateneu	Ceará	Brazil
Ana Laura Brandão	Fundação Oswaldo Cruz	Rio de Janeiro	Brazil
Ana Lúcia Schaefer Ferreira de Mello	Universidade Federal de Santa Catarina	Santa Catarina	Brazil
Ana Lúcia Lyrio de Oliveira	Universidade de São Paulo	São Paulo	Brazil
Ana Maria Roselino	Universidade de São Paulo	São Paulo	Brazil
Ana Marli Christovam Sartori	Universidade de São Paulo	São Paulo	Brazil
Ana Patrícia Alves da Silva	Universidade de Fortaleza	Ceará	Brazil
Ana Paula da Costa Marques	Universidade Federal de Mato Grosso do Sul	Mato Grosso do Sul	Brazil
Ana Paula da Cunha	Fundação Oswaldo Cruz	Rio de Janeiro	Brazil
Ana Paula de Rocha Sales Miranda	Universidade Federal da Paraíba	Paraíba	Brazil
André Luis Alves de Quevedo	Secretaria Estadual de Saúde do Rio Grande do Sul	Rio Grande do Sul	Brazil
André Luiz Bigal	Universidade Federal de São Paulo	São Paulo	Brazil
André Luiz Machado das Neves	Universidade do Estado do Amazonas	Amazonas	Brazil
Andrea Moreira Arrué	Instituto Federal do Paraná	Paraná	Brazil
Andréa Tenório Correia da Silva	Universidade de São Paulo	São Paulo	Brazil
Andréia Insabralde de Queiroz-Cardoso	Universidade Federal de Mato Grosso do Sul	Mato Grosso do Sul	Brazil
Andrezza Santiago	Universidade Federal de Lavras	Minas Gerais	Brazil
Ângela Scalabrin	Santa Casa de São Paulo	São Paulo	Brazil
Angélica Atala Lombelo Campos	Universidade Federal de Juiz de Fora	Minas Gerais	Brazil
Angélica Ribeiro e Silva	Universidade Federal de Minas Gerais	Minas Gerais	Brazil
Angélica Yukari Takemoto	Centro Universitário Guairacá	Paraná	Brazil
Anke Bergmann	Instituto Nacional do Câncer	Rio de Janeiro	Brazil
Ann Jansen	Universidade Federal de Minas Gerais	Minas Gerais	Brazil
Antonio Augusto Ferreira Carioca	Universidade de Fortaleza	Ceará	Brazil
Antônio Carlos Frias	Universidade de São Paulo	São Paulo	Brazil
Antônio Carlos Vieira Ramos	Universidade do Estado de Minas Gerais	Minas Gerais	Brazil
Antonio da Silva Menezes Junior	Universidade Federal de Goiás	Goiás	Brazil
Antonio Gasparoto	Universidade Federal de Mato Grosso do Sul	Mato Grosso do Sul	Brazil
Antonio Ygor Modesto de Oliveira	Ministério da Saúde	Distrito Federal	Brazil
Ariela Mota Ferreira	Universidade Estadual de Montes Claros	Minas Gerais	Brazil
Ariene Carmo	Universidade Federal de Minas Gerais	Minas Gerais	Brazil
Arn Migowski Rocha dos Santo	Instituto Nacional do Câncer	Rio de Janeiro	Brazil
Arthur Duarte Fantesia Costa Cruz	Secretaria de Estado de Saúde de Mato Grosso do Sul	Mato Grosso do Sul	Brazil
Bárbara dos Santos Simões	Universidade Professor Edson Antônio Velano	Minas Gerais	Brazil
Betina Berlitz	Serviço Social do Comércio	Rio Grande do Sul	Brazil
Betine Pinto Moehlecke Iser	Universidade do Sul de Santa Catarina	Santa Catarina	Brazil
Breno de Oliveira Ferreira	Universidade Federal do Amazonas	Amazonas	Brazil
Brigina Kemp	Conselho de Secretários Municipais de Saúde do Estado de São Paulo	São Paulo	Brazil
Bruna Drumond Silveira	Fundação Oswaldo Cruz	Rio de Janeiro	Brazil
Bruna Moretti Luchesi	Universidade Federal de Mato Grosso do Sul	Mato Grosso do Sul	Brazil
Bruna Teles Soares Beserra	Universidade Federal de Minas Gerais	Minas Gerais	Brazil
Cacilda Tezelli Junqueira Padovani	Universidade Federal de Mato Grosso do Sul	Mato Grosso do Sul	Brazil
Caio Graco Zeppelini	Universidade Federal da Bahia	Bahia	Brazil
Caio Vieira de Barros Arato	Universidade Estadual de Campinas	São Paulo	Brazil
Carla Fabiana Tenanni	Faculdade de Medicina de Jundiaí	São Paulo	Brazil
Carla Rocha Pereira	Fundação Oswaldo Cruz	Rio de Janeiro	Brazil
Carla Luzia França Araújo	Universidade Federal do Rio de Janeiro	Rio de Janeiro	Brazil
Carlos Frederico S. Marques	Fundação Faculdade de Medicina	São Paulo	Brazil
Carlos José de Paula Silva	Universidade Federal de Minas Gerais	Minas Gerais	Brazil
Carmen Silvia Bruniera Domingues	Secretaria de Estado da Saúde de São Paulo	São Paulo	Brazil
Carolina Abreu Henn de Araújo	Universidade Federal de Santa Catarina	Santa Catarina	Brazil
Carolina de Campos Carvalho	Fundação Oswaldo Cruz	Rio de Janeiro	Brazil
Carolina Mariano Pompeo	Universidade Federal de Mato Grosso do Sul	Mato Grosso do Sul	Brazil
Carolina Muller Ferreira	Universidade Estadual de Campinas	São Paulo	Brazil
Caroline de Barros Gomes	Universidade Estadual Paulista	São Paulo	Brazil
Cássia Barbosa Reis	Universidade Estadual do Mato Grosso do Sul	Mato Grosso do Sul	Brazil
Cássia CP Mendicino	Secretaria de Estado de Saude de Minas Gerais	Minas Gerais	Brazil
Cassio Hartmann	Instituto Federal de Alagoas	Alagoas	Brazil
Cátia Regina Ficagna	Universidade Federal do Rio Grande do Sul	Rio Grande do Sul	Brazil
Catiane Costa Viana	Universidade Federal de Minas Gerais	Minas Gerais	Brazil
Christine Faustino	Universidade Estadual do Mato Grosso do Sul	Mato Grosso do Sul	Brazil
Cintia de Freitas Oliveira	Instituto de Saúde	São Paulo	Brazil
Clareci Silva Cardoso	Universidade Federal de São João del-Rei	Minas Gerais	Brazil
Cláudia Carolina Costa Braga	Universidade Estadual Paulista	São Paulo	Brazil
Claudia Cristina Lima de Castro	Secretaria de Saúde do Recife	Pernambuco	Brazil
Cláudia Raulino Tramontt	Universidade de São Paulo	São Paulo	Brazil
Claudia Tavares Regadas	Fundação Oswaldo Cruz	Rio de Janeiro	Brazil
Cláudia Volpe Chaves	Hospital Regional de Mato Grosso do Sul	Mato Grosso do Sul	Brazil
Claudio Gruber Mann	Universidade Federal do Rio de Janeiro	Rio de Janeiro	Brazil
Clineu de Mello Almada Filho	Universidade Federal de São Paulo	São Paulo	Brazil
Cristiane Helena Gallasch	Universidade do Estado do Rio de Janeiro	Rio de Janeiro	Brazil
Cristianny Miranda	Universidade Federal de Minas Gerais	Minas Gerais	Brazil
Cristiano Bertolossi Marta	Universidade do Estado do Rio de Janeiro	Rio de Janeiro	Brazil
Cristina Pimenta	Instituto Nacional de Infectologia Evandro Chagas(INI), Fiocruz	Rio de Janeiro	Brazil
Cristina Wide Pissetti	Universidade Federal da Paraíba	Paraíba	Brazil
Cristine Vieira do Bonfim	Fundação Joaquim Nabuco	Pernambuco	Brazil
Dalila de Carvalho Silva Gonzaga	Universidade de Brasília	Distrito Federal	Brazil
Daniel Hideki Bando	Universidade Federal de Alfenas	Minas Gerais	Brazil
Daniela de Almeida Pereira	Universidade Federal de Viçosa	Minas Gerais	Brazil
Danúbia Hillesheim	Universidade Federal de Santa Catarina	Santa Catarina	Brazil
Danyllo do Nascimento Silva Junior	Universidade Federal do Rio Grande do Norte	Rio Grande do Norte	Brazil
Dartagnan Pinto Guedes	Universidade Estadual do Norte do Paraná	Paraná	Brazil
David Coelho	Harvard University	Massachusets	Estados Unidos
Dayanne da Costa Maynard	Centro Universitário de Brasília	Distrito Federal	Brazil
Dayse Sanches Guimarães Paião	Universidade Federal da Grande Dourados	Mato Grosso do Sul	Brazil
Débora Castanheira	Fundação Oswaldo Cruz	Rio de Janeiro	Brazil
Débora Driemeyer Wilbert	Universidade Santo Amaro	São Paulo	Brazil
Denis Gonçalves Ferreira	Centro Universitário Várzea Grande	Mato Grosso	Brazil
Denise Eliziana de Souza	Escola Nacional de Saúde Pública Rio de Janeiro	Rio de Janeiro	Brazil
Denise Fortes	Secretaria de Estado de Saúde de Mato Grosso do Sul	Mato Grosso do Sul	Brazil
Deyvyd Manoel Condé Andrade	Secretaria Municipal de Saúde do Rio de Janeiro	Rio de Janeiro	Brazil
Diva Furtado Lacerda	Universidade Federal de Goiás	Goiás	Brazil
Divanise Suruagy Correia	Universidade Federal de Alagoas	Alagoas	Brazil
Doroteia Hofelmann	Universidade Federal do Paraná	Paraná	Brazil
Ed Wilson Rodrigues Vieira	Universidade Federal de Minas Gerais	Minas Gerais	Brazil
Edgar Oshiro	Escola de Saúde Pública Dr. Jorge David Nasser	Mato Grosso do Sul	Brazil
Eduardo Hage Carmo	Fundação Oswaldo Cruz	Distrito Federal	Brazil
Eduardo Jorge da Fonseca Lima	Instituto de Medicina Integral Professor Fernando Figueira	Pernambuco	Brazil
Elen Ferraz Teston	Universidade Federal de Mato Grosso do Sul	Mato Grosso do Sul	Brazil
Eliane Rolim de Holanda	Universidade Federal da Paraíba	Paraíba	Brazil
Eliete Albano de Azevedo Guimarães	Universidade Federal de São João del-Rei	Minas Gerais	Brazil
Elisa Prezotto Giordani	Universidade de São Paulo	São Paulo	Brazil
Elisabete Pereira Silva	Universidade Federal de Pernambuco	Pernambuco	Brazil
Elisabeth Meloni Vieira	Universidade de São Paulo	São Paulo	Brazil
Elisângela Aparecida da Silva Lizzi	Universidade Tecnológica Federal do Paraná	Paraná	Brazil
Elisete Duarte	Universidade Federal de Mato Grosso	Mato Grosso	Brazil
Eliseu Alves Waldman	Universidade de São Paulo	São Paulo	Brazil
Emile Danielly Amorim Pereira	Faculdade Edufor	Maranhão	Brazil
Emilia Moreira Jalil	Fundação Oswaldo Cruz	Rio de Janeiro	Brazil
Erick Soares Lisboa	Universidade Federal da Bahia	Bahia	Brazil
Erlandson Ferreira Saraiva	Universidade Federal de Mato Grosso do Sul	Mato Grosso do Sul	Brazil
Erly Catarina de Moura	Universidade de Brasília	Distrito Federal	Brazil
Ernani Tiaraju de Santa Helena	Universidade Regional de Blumenau	Santa Catarina	Brazil
Ester Souto	Fundação Oswaldo Cruz	Rio de Janeiro	Brazil
Eunice Francisca Martins	Universidade Federal de Minas Gerais	Minas Gerais	Brazil
Evelina Chapman	Fundação Oswaldo Cruz	Distrito Federal	Brazil
Expedito José de Albuquerque Luna	Universidade de São Paulo	São Paulo	Brazil
Fabiana Alves Soares	Universidade Federal do Maranhão	Maranhão	Brazil
Fabiana Baggio Nerbass	Fundação Pró-Rim	Santa Catarina	Brazil
Fabiana Schuelter-Trevisol	Universidade do Sul de Santa Catarina	Santa Catarina	Brazil
Fabiane Letícia de Freitas	Universidade de São Paulo	São Paulo	Brazil
Fabrizia Foletto	Universidade Federal de Mato Grosso do Sul	Mato Grosso do Sul	Brazil
Felipe Guimarães Tavares	Universidade Federal Fluminense	Rio de Janeiro	Brazil
Felipe Teixeira de Mello Freitas	Universidade do Distrito Federal	Distrito Federal	Brazil
Fernanda Esthefane Garrides Oliveira	Universidade Federal de Minas Gerais	Minas Gerais	Brazil
Fernanda Leticia Ferreira	Fundação Oswaldo Cruz	Rio de Janeiro	Brazil
Fernanda Marcelina Silva	Universidade de São Paulo	São Paulo	Brazil
Fernando Antônio Ribeiro de Gusmão-filho	Universidade de Pernambuco	Pernambuco	Brazil
Fernando Lopes Tavares de Lima	Instituto Nacional do Câncer	Rio de Janeiro	Brazil
Fernando Silva Guimarães	Universidade Federal de Pelotas	Rio Grande do Sul	Brazil
Flávia Casale Abe	Universidade de Sorocaba	São Paulo	Brazil
Flavia Aparecida Dias Marmo	Universidade Federal do Triangulo Mineiro	Minas Gerais	Brazil
Flávia Meneguetti Pieri	Universidade Estadual de Londrina	Paraná	Brazil
Flávia Mori Sarti	Universidade de São Paulo	São Paulo	Brazil
Flávia Reis de Andrade	Universidade de Brasília	Distrito Federal	Brazil
Flávio Renato Reis de Moura	Universidade La Salle	Rio Grande do Sul	Brazil
Franciele Costa da Silva Perez	Universidade Estadual Paulista	São Paulo	Brazil
Francilene Jane Pereira	Universidade Federal da Paraiba	Paraíba	Brazil
Francisco Inácio Pinkusfeld Bastos	Fundação Oswaldo Cruz	Rio de Janeiro	Brazil
Francisco Tustumi	Universidade de São Paulo	São Paulo	Brazil
Gabriel Pavinati	Universidade Estadual de Maringá	Paraná	Brazil
Gabriel Zorello Laporta	Centro Universitário da Faculdade de Medicina do ABC	São Paulo	Brazil
Gabriela Maria Cavalcanti Costa	Universidade Estadual da Paraíba	Paraíba	Brazil
Gabriela Soledad Márdero García	Universidade Federal do Ceara	Ceará	Brazil
Gabriella Morais Duarte Miranda	Universidade Federal de Pernambuco	Pernambuco	Brazil
Galba Freire Moita	Ministério da Saúde	Distrito Federal	Brazil
George Jó Bezerra Sousa	Ministério da Saúde	Distrito Federal	Brazil
Gerson Barbosa	Instituto Pasteur de São Paulo	São Paulo	Brazil
Gerson Luiz Marinho	Universidade Federal do Rio de Janeiro	Rio de Janeiro	Brazil
Giselle Freitas	Universidade Federal de Minas Gerais	Minas Gerais	Brazil
Giulia Silva	Universidade de São Paulo	São Paulo	Brazil
Glaucia Maria Bressan	Universidade Tecnológica Federal do Paraná	Paraná	Brazil
Gláucio de Oliveira Nangino	Hospital da Polícia Militar de Minas Gerais	Minas Gerais	Brazil
Guilherme Diogo Silva	Universidade de São Paulo	São Paulo	Brazil
Gustavo Magno Baldin Tiguman	Universidade Estadual de Campinas	São Paulo	Brazil
Gustavo Santa Roza Saggese	Universidade de São Paulo	São Paulo	Brazil
Haroldo da Silva Ferreira	Universidade Federal de Alagoas	Alagoas	Brazil
Heitor Andrade	Universidade de São Paulo	São Paulo	Brazil
Heitor Victor Veiga da Costa	Universidade Federal de Pernambuco	Pernambuco	Brazil
Helena Ribeiro	Universidade de São Paulo	São Paulo	Brazil
Helierson Gomes	Universidade Federal do Norte do Tocantins	Tocantins	Brazil
Iana Mundim de Oliveira	Universidade Federal de Goiás	Goiás	Brazil
Icaro José Santos Ribeiro	Universidade Estadual de Santa Cruz	Bahia	Brazil
Igor Gonçalves Ribeiro	Ministério da Saúde	Distrito Federal	Brazil
Inara Cunha	Escola de Saúde Pública Dr. Jorge David Nasser	Mato Grosso do Sul	Brazil
Indianara Maria de Barros Canuto	Secretaria de Saúde do Recife	Pernambuco	Brazil
Inês Rugani Ribeiro de Castro	Universidade do Estado do Rio de Janeiro	Rio de Janeiro	Brazil
Inês Dourado	Universidade Federal da Bahia	Bahia	Brazil
Isabela Thaís Machado de Jesus	Universidade Federal de São Carlos	São Paulo	Brazil
Isabele Barboza Moura	Fundação Oswaldo Cruz	Rio de Janeiro	Brazil
Isabella da Motta Passos	Universidade do Estado do Amazonas	Amazonas	Brazil
Isabella Vitral Pinto	Instituto René Rachou	Minas Gerais	Brazil
Isadora Caixeta da Silveira Ferreira	Universidade Federal de Uberlândia	Minas Gerais	Brazil
Isis Carvalho	Ministério da Saúde	Distrito Federal	Brazil
Italo Wesley Oliveira Aguiar	Universidade Federal do Ceará	Ceará	Brazil
Jack Roberto Silva Fhon	Universidade de São Paulo	São Paulo	Brazil
Jacqueline de Almeida Goncalves Sachett	Universidade do Estado do Amazonas	Amazonas	Brazil
Janaina Fonseca Almeida Souza	Universidade Federal de Minas Gerais	Minas Gerais	Brazil
Jaqueline Arboit	Universidade Federal de Santa Maria	Rio Grande do Sul	Brazil
Jaqueline Dario Capobiango	Universidade La Salle	Rio Grande do Sul	Brazil
Jaqueline Gomes de Jesus	Instituto Federal do Rio de Janeiro	Rio de Janeiro	Brazil
Jardel Corrêa de Oliveira	Prefeitura Municipal de Florianópolis	Santa Catarina	Brazil
Jardeliny Corrêa de Penha	Universidade Federal do Ceará	Ceará	Brazil
Jean Ezequiel Limongi	Universidade Federal de Uberlândia	Minas Gerais	Brazil
Jeferson Agnelli	Universidade da Sorocaba	São Paulo	Brazil
Jesem Douglas Yamall Orellana	Leônidas e Maria Deane Institute Fiocruz Amazonas	Amazonas	Brazil
Jéssica Muzy Rodrigues	Fundação Oswaldo Cruz	Rio de Janeiro	Brazil
Jessica Rasquim Araujo	Universidade Federal de Minas Gerais	Minas Gerais	Brazil
Jessye Melgarejo do Amaral Giordani	Universidade Federal de Santa Maria	Rio Grande do Sul	Brazil
João Alberto Martins Rodrigues	Universidade Federal do Paraná	Paraná	Brazil
João Carlos Alchieri	Universidade Federal do Rio Grande do Norte	Rio Grande do Norte	Brazil
João Paulo Cola	Secretaria de Estado da Saúde do Espírito Santo	Espírito Santo	Brazil
Johnatan Martins Sousa	Universidade Federal de Goiás	Goiás	Brazil
Jonas Eduardo Monteiro dos Santos	Instituto Nacional do Câncer	Rio de Janeiro	Brazil
Jonas Loiola Gonçalves	Universidade Estadual do Ceará	Ceará	Brazil
José Eduardo Gomes Arruda	Universidade Federal do Pará	Pará	Brazil
José Eleutério Junior	Universidade Federal do Ceará	Ceará	Brazil
José Fernando Salvador Carrillo	Universidade de Sorocoba	São Paulo	Brazil
José Geraldo Leite Ribeiro	Faculdade de Ciências Médicas de Minas Gerais	Minas Gerais	Brazil
José Rodolfo Lucena	Faculdade Medicina do Sertão	Pernambuco	Brazil
Josy Maria de Pinho da Silva	Organização Pan-Americana da Saúde	Distrito Federal	Brazil
Julia Gomes Freitas	Universidade de São Paulo	São Paulo	Brazil
Juliana Fernandes Kabad	Universidade Federal do Mato Grosso	Mato Grosso	Brazil
Juliana Gonçalves Reis	Fundação Oswaldo Cruz	Rio de Janeiro	Brazil
Juliana Lustosa Torres	Universidade Federal de Minas Gerais	Minas Gerais	Brazil
Juliana Mara Flores Bicalho	Universidade Federal de São João del-Rei	Minas Gerais	Brazil
Juliana Pavan Zuliani	Fundação Oswaldo Cruz	Rondônia	Brazil
Juliana Quero Reimão	Faculdade de Medicina de Jundiaí	São Paulo	Brazil
Juliana Vallim Jorgetto Santos	Universidade Federal de São Paulo	São Paulo	Brazil
Junir Antonio Lutinski	Universidade Comunitária da Região de Chapecó	Santa Catarina	Brazil
Kaciane Corrêa Mochizuke	Secretaria de Saúde de Porto Murtinho	Mato Grosso do Sul	Brazil
Karin Romano Posseger	Universidade Federal do Ceará	Ceará	Brazil
Karine Ferreira Barbosa	Secretaria de Estado de Saúde de Mato Grosso do Sul	Mato Grosso do Sul	Brazil
Kátia Cristina Barbosa Ferreira	Universidade de Pernambuco	Pernambuco	Brazil
Katiusse Rezende Alves	Universidade Federal de Viçosa	Minas Gerais	Brazil
Keitty Regina Cordeiro de Andrade	Universidade de Brasília	Distrito Federal	Brazil
Kelly Alves Magalhães	Universidade Federal de Minas Gerais	Minas Gerais	Brazil
Kelly Cristina do Nascimento	Universidade de Pernambuco	Pernambuco	Brazil
Kellyn Kessiene de Sousa Cavalcante	Universidade Federal do Ceará	Ceará	Brazil
Kérilin Stancine Santos Rocha	Universidade Federal do Espírito Santo	Espírito Santo	Brazil
Laio Magno	Universidade do Estado da Bahia	Bahia	Brazil
Larissa Fortunato Araujo	Universidade Federal do Ceara	Ceará	Brazil
Larissa Loures Mendes	Universidade Federal de Minas Gerais	Minas Gerais	Brazil
Larissa Zepka Baumgarten Rodrigues	Fundação de apoio à saúde de Curitiba	Paraná	Brazil
Laura Inês Maricato Otani	Universidade de Sorocoba	São Paulo	Brazil
Lauro Ferreira da Silva Pinto Neto	Santa Casa de Vitória	Espírito Santo	Brazil
Laylla Ribeiro Macedo	Universidade Federal do Rio de Janeiro	Rio de Janeiro	Brazil
Leandro Aparecido Souza	Universidade de Sorocoba	São Paulo	Brazil
Lenir Aparecida Chaves	Universidade Federal de Minas Gerais	Minas Gerais	Brazil
Leonardo Trench	Universidade de São Paulo	São Paulo	Brazil
Letícia Russo	Universidade Estadual de Maringá	Paraná	Brazil
Lia Borges de Mattos Custodio	Universidade Estadual Paulista	São Paulo	Brazil
Liandro da Cruz Lindner	Faculdade das Américas	São Paulo	Brazil
Lidia Bezerra Barbosa	Universidade Federal de Alagoas	Alagoas	Brazil
Lidiane da Silveira Gouvea Toledo	Fundação Oswaldo Cruz	Bahia	Brazil
Lidiane Sousa	Universidade Federal de Minas Gerais	Minas Gerais	Brazil
Lígia Amparo	Universidade Federal da Bahia	Bahia	Brazil
Lilian Cristina Rezende	Universidade Federal de Minas Gerais	Minas Gerais	Brazil
Lílian Fernandes Amarante	Fundação Oswaldo Cruz Ceará	Ceará	Brazil
Lissandra Santos Degner	Universidade Federral da Bahia	Bahia	Brazil
Lívia de Lima Moura	Secretaria Municipal de Saúde do Rio de Janeiro	Rio de Janeiro	Brazil
Luana da Silva Baptista Arpini	Secretaria Estadual de Saúde do Espírito Santo	Espírito Santo	Brazil
Luana Silva Rodrigues de Araujo	Hospital Israelita Albert Einstein	São Paulo	Brazil
Lucas Vinícius de Lima	Universidade Estadual de Maringá	Paraná	Brazil
Lucélia Colares	Universidade Federal do Rio de Janeiro	Rio de Janeiro	Brazil
Lucélia dos Santos Silva	Hospital Albert Einstein	São Paulo	Brazil
Luciana Bihain Hagemann de Malfussi	Universidade Federal de Santa Catarina	Santa Catarina	Brazil
Luciana Freire de Carvalho	Universidade Federal do Rio de Janeiro	Rio de janeiro	Brazil
Luciana Martins Rozman	Universidade São Judas Tadeu	São Paulo	Brazil
Lucieni Conterno	Universidade Estadual de Campinas	São Paulo	Brazil
Luis Phillipe Nagem Lopes	Universidade do Estado do Rio de Janeiro	Rio de Janeiro	Brazil
Luísa Silveira da Silva	Universidade Federal de Pelotas	Rio Grande do Sul	Brazil
Luiz Antonio Bastos Camacho	Fundação Oswaldo Cruz	Rio de janeiro	Brazil
Manoel Carlos Sampaio de Almeida Ribeiro	Universidade Presbiteriana Mackenzie	São Paulo	Brazil
Marcela de Abreu Moniz	Universidade Federal Fluminense	Rio de Janeiro	Brazil
Marcelo Coelho Goiato	Universidade Estadual Paulista Júlio de Mesquita Filho	São Paulo	Brazil
Márcia Cristina Dalla Costa	Universidade Estadual do Oeste do Paraná	Paraná	Brazil
Marco Aurélio Palazzi Sáfadi	Santa Casa de São Paulo	São Paulo	Brazil
Marcos Augusto Moraes Arcoverde	Universidade Estadual do Oeste do Paraná	Paraná	Brazil
Marcos Cláudio Signorelli	Universidade Federal do Paraná	Paraná	Brazil
Marcos Pereira Santos	Universidade Federal da Bahia	Bahia	Brazil
Maria Alves	Secretaria Municipal de Saude de Dois Irmãos do Buriti	Mato Grosso do Sul	Brazil
Maria Amélia Veras	Santa Casa de São Paulo	São Paulo	Brazil
Maria Angélica Tavares de Medeiros	Universidade Federal de São Paulo	São Paulo	Brazil
Maria Auxiliadora Martins	Universidade Federal de Minas Gerais	Minas Gerais	Brazil
Maria Beatriz Kneipp Dias	Instituto Nacional do Câncer	Rio de Janeiro	Brazil
Maria Cecília Ramos de Carvalho	Universidade Federal de Minas Gerais	Minas Gerais	Brazil
Maria Cristina Antunes Willemann	Conselho de Secretarias Municipais de Saúde de Santa Catarina	Santa Catarina	Brazil
Maria Cristina Mazzaia	Universidade Federal de São Paulo	São Paulo	Brazil
Maria Cynthia Braga	Instituto Aggeu Magalhães	Recife	Brazil
Maria do Carmo Fernandez Lourenço Haddad	Universidade Estadual de Londrina	Paraná	Brazil
Maria Laura Louzada	Universidade de São Paulo	São Paulo	Brazil
Maria Lúcia Zanetti	Universidade de São Paulo	São Paulo	Brazil
Maria Luiza Ferreira Imburana da Silva	Universidade Federal de Pernambuco	Pernambuco	Brazil
Maria Rita Donalísio	Universidade Estadual de Campinas	São Paulo	Brazil
Mariana Aparecida Brozoski	Universidade de São Paulo	São Paulo	Brazil
Mariana Gabriel	Universidade de São Paulo	São Paulo	Brazil
Mariana Lopes Galante	Universidade de São Paulo	São Paulo	Brazil
Mariana Souza Lopes	Universidade Federal da Paraíba	Paraíba	Brazil
Marilia Mastrocolla de Almeida Cardoso	Ministério da Saúde	Distrito Federal	Brazil
Mário Círio Nogueira	Universidade Federal de Juiz de Fora	Minas Gerais	Brazil
Mario Jorge Sobreira da Silva	Instituto Nacional do Câncer	Rio de Janeiro	Brazil
Maristela Honório Cayetano	Universidade de Mogi das Cruzes	São Paulo	Brazil
Marlene Azevedo Magalhães Monteiro	Universidade Federal de Minas Gerais	Minas Gerais	Brazil
Marta Heloisa Lopes	Universidade de São Paulo	São Paulo	Brazil
Maryana Carmello da Costa	Universidade de São Paulo	São Paulo	Brazil
Matheus Santos Melo	Universidade Federal de Sergipe	Sergipe	Brazil
Mayara Nascimento de Vasconcelos	Universidade Estadual do Ceará	Ceará	Brazil
Mayara Secco Torres da Silva	Fundação Oswaldo Cruz	Rio de Janeiro	Brazil
Melissa Luciana de Araújo	Universidade Federal de Minas Gerais	Minas Gerais	Brazil
Mercedes de Oliveira Neto	Universidade do Estado do Rio de Janeiro	Rio de janeiro	Brazil
Michele Fernanda Borges da Silva	Fundação Oswaldo Cruz	Rio de Janeiro	Brazil
Michelle Ramos	Fundação Oswaldo Cruz	Rio de Janeiro	Brazil
Milton Luiz Gorzoni	Santa Casa de São Paulo	São Paulo	Brazil
Naiara Sperandio	Universidade Federal do Rio de Janeiro	Rio de Janeiro	Brazil
Nair Tavares Milhem Ygnatios	Universidade Federal de Minas Gerais	Minas Gerais	Brazil
Natacha Kalline de Oliveira	Universidade de São Paulo	São Paulo	Brazil
Natacha Toral	Universidade de Brasília	Distrito Federal	Brazil
Natália Daroit	Universidade Federal do Rio Grande do Sul	Rio Grande do Sul	Brazil
Nathália Guimarães	Universidade Federal de Minas Gerais	Minas Gerais	Brazil
Nathália Luíza Ferreira	Universidade Federal de Lavras	Minas Gerais	Brazil
Nathan Aratani	Universidade Federal de Mato Grosso do Sul	Mato Grosso do Sul	Brazil
Neide Aparecida Boldrini	Universidade Federal do Espírito Santo	Espírito Santo	Brazil
Nicole Pischel	Universidade de São Paulo	São Paulo	Brazil
Norma Suely de Oliveira Farias	Instituto de Saúde	São Paulo	Brazil
Osmar de Oliveira Cardoso	Universidade Federal do Piauí	Piauí	Brazil
Oswaldo Villa Campos	Universidade de São Paulo	São Paulo	Brazil
Oziris Simões	Santa Casa de São Paulo	São Paulo	Brazil
Pablo Cardozo Rocon	Universidade Federal de Mato Grosso	Mato Grosso	Brazil
Pamela Alejandra Saavedra	Conselho Federal de Farmácia	Distrito Federal	Brazil
Pantelis Varvaki Rados	Universidade Federal do Rio Grande do Sul	Rio Grande do Sul	Brazil
Paola Barbosa Marchesini	Ministério da Saúde	Distrito Federal	Brazil
Patricia Jaime	Universidade de São Paulo	São Paulo	Brazil
Patrícia Maria Oliveira Machado	Universidade Federal de Santa Catarina	Santa Catarina	Brazil
Patrícia Pinheiro de Freitas	Universidade Federal dos Vales do Jequitinhonha e Mucuri	Minas Gerais	Brazil
Pauline Lorena Kale	Universidade Federal do Rio de Janeiro	Rio de Janeiro	Brazil
Paulo Carrara de Castro	Santa Casa de Misericórdia de São Paulo	São Paulo	Brazil
Paulo Henrique Faria Domingues	Universidade de Brasília	Distrito Federal	Brazil
Pedro Arthur Araújo	Ministério da Saúde	Distrito Federal	Brazil
Pedro Câncio Neto	Universidade de São Paulo	São Paulo	Brazil
Pedro Castro	Pontifícia Universidade Católica	Rio de Janeiro	Brazil
Pedro Henrique Brito da Silva	Secretaria Municipal de Saúde de Senador Canedo	Goiás	Brazil
Policardo Gonçalves da Silva	Universidade do Estado de Minas Gerais	Minas Gerais	Brazil
Priscila de Oliveira Luz	Universidade de São Paulo	São Paulo	Brazil
Rafael Aiello Bomfim	Universidade Federal de Mato Grosso do Sul	Mato Grosso do Sul	Brazil
Rafael Claro	Universidade Federal de Minas Gerais	Minas Gerais	Brazil
Rafaela Soares Rech	Universidade Federal de Ciências da Saúde de Porto Alegre	Rio Grande do Sul	Brazil
Raphael de Freitas Saldanha	Fundação Oswaldo Cruz	Rio de Janeiro	Brazil
Raquel Aparecida Ferreira	Instituto René Rachou	Minas Gerais	Brazil
Raquel de Deus Mendonça	Universidade Federal de Ouro Preto	Minas Gerais	Brazil
Raquel Silveira Bello Stucchi	Universidade Estadual de Campinas	São Paulo	Brazil
Rayane Cavalcante Pereira Batista	Universidade de Brasília	Distrito Federal	Brazil
Raysa Álvarez	Universidade de São Paulo	São Paulo	Brazil
Regina Marques	Prefeitura do Município de São Paulo	São Paulo	Brazil
Regina Tamaki	Universidade de São Paulo	São Paulo	Brazil
Renata Adrielle Lima Vieira	Universidade Federal da Paraíba	Paraíba	Brazil
Renata Cordeiro Domingues	Faculdade Pernambucana de Saúde	Pernambuco	Brazil
Renata Paz Leal Pereira	Universidade de São Paulo	São Paulo	Brazil
Renato Kfouri	Sociedade Brasileira de Imunizações	São Paulo	Brazil
Reyce Koga	Secretaria Municipal de Saúde de Manaus	Amazonas	Brazil
Ricardo Gadelha de Abreu	Ministério da Saúde	Distrito Federal	Brazil
Ricardo José de Paula Souza e Guimarães	Instituto Evandro Chagas	Pará	Brazil
Roberta Costa Jorge	Universidade do Estado do Rio de Janeiro	Rio de Janeiro	Brazil
Roberta Pereira Niquini	Instituto Federal do Rio de Janeiro	Rio de Janeiro	Brazil
Rodrigo Citton Padilha dos Reis	Ministério da Saúde	Distrito Federal	Brazil
Rodrigo Pinheiro de Toledo Vianna	Universidade Federal da Paraíba	Paraíba	Brazil
Rosa Maria Soares Madeira Domingues	Fundação Oswaldo Cruz	Rio de Janeiro	Brazil
Rosalia Garcia Neves	Secretaria Estadual de Saúde do Rio Grande do Sul	Rio Grande do Sul	Brazil
Rosana Richtmann	Instituto Emílio Ribas	São Paulo	Brazil
Samuel Benjoino Ferraz Aquino	Universidade Federal de Mato Grosso do Sul	Mato Grosso do Sul	Brazil
Samuel Lopes dos Santos	Universidade Federal do Piauí	Piauí	Brazil
Sandra Brignol	Universidade Federal Fluminense	Rio de Janeiro	Brazil
Sandra Maria do Valle Leone de Oliveira	Fundação Oswaldo Cruz	Mato Grosso do Sul	Brazil
Sarah Arangurem Karam	Universidade Católica de Pelotas	Rio Grande do Sul	Brazil
Sávio Marcelino Gomes	Universidade Federal da Paraíba	Paraíba	Brazil
Sheilla Siedler Tavares	Centro Universitário Facens	São Paulo	Brazil
Sheyla Maria Leite	Ministério da Saúde	Distrito Federal	Brazil
Sidiany Mendes Pimentel	Instituto Federal do Tocantins	Tocantins	Brazil
Silvia Helena Mendonça de Moraes	Fundação Oswaldo Cruz	Mato Grosso do Sul	Brazil
Silvio Barberato-Filho	Universidade de Sorocoba	São Paulo	Brazil
Sonia Silvestrin	Secretaria Municipal de Saúde de Porto Alegre	Rio Grande do Sul	Brazil
Soraya S Smaili	Universidade Federal de São Paulo	São Paulo	Brazil
Stela Lemos	Universidade Federal de Minas Gerais	Minas Gerais	Brazil
Tânia Cristina de Mattos Barros Petraglia	Centro de Referência para Imunobiológicos Especiais Myrtes Amorelli Gonzaga	Rio de janeiro	Brazil
Tania Maria de Araujo	Universidade Estadual de Feira de Santana	Bahia	Brazil
Tatiana Resende Prado Rangel Oliveira	Pontifícia Universidade Católica de Minas Gerais	Minas Gerais	Brazil
Tatiana Sadalla Collese	Centro Universitário São Camilo	São Paulo	Brazil
Tayanny Margarida Menezes Almeida Biase	Universidade Estadual de Campinas	São Paulo	Brazil
Teresa Cristina Vieira Segatto	Secretaria de Saúde do Distrito Federal	Distrito Federal	Brazil
Thais Gularte Della Vechia	Universidade Federal de Pelotas	Rio Grande do Sul	Brazil
Thais Machado	Universidade de São Paulo	São Paulo	Brazil
Thaynã Ramos Flores	Universidade Federal de Pelotas	Rio Grande do Sul	Brazil
Thayssa Maluff de Mello	Universidade Federal de Mato Grosso do Sul	Mato Grosso do Sul	Brazil
Thereza Maria Magalhães Moreira	Universidade Estadual do Ceará	Ceará	Brazil
Thiago Torres	Fundação Oswaldo Cruz	Rio de Janeiro	Brazil
Valéria de Campos Orsi	Universidade de Sorocoba	São Paulo	Brazil
Valéria Valim	Universidade Federal do Espírito Santo	Espírito Santo	Brazil
Vandilson Pinheiro Rodrigues	Universidade Federal do Maranhão	Maranhão	Brazil
Vanessa Alves Ferreira	Universidade Federal de Minas Gerais	Minas Gerais	Brazil
Vanessa Coelho de Aquino Benjoino Ferraz	Secretaria Municipal de Saúde de Campo Grande	Mato Grosso do Sul	Brazil
Vanessa Cruz Santos	Universidade Federal do Rio de Janeiro	Rio de Janeiro	Brazil
Vanessa Gomes Lima	Universidade Estadual de Campinas	São Paulo	Brazil
Vanessa Moraes Bezerra	Universidade Federal da Bahia	Bahia	Brazil
Vanessa Ramos Kirsten	Universidade Federal de Santa Maria	Rio Grande do Sul	Brazil
Vânia Paula Stolte-Rodrigues	Universidade Federal de Mato Grosso do Sul	Mato Grosso do Sul	Brazil
Vanner Boere Souza	Universidade Federal do Sul da Bahia	Bahia	Brazil
Vicente Sperb Antonello	Hospital Fêmina	Rio Grande do Sul	Brazil
Vinícius Gonçalves de Paula	Universidade Federal de Minas Gerais	Minas Gerais	Brazil
Virgina Weffort	Universidade Federal do Triangulo Mineiro	Minas Gerais	Brazil
Virgínia D. Carvalho	Universidade Federal de Alfenas	Minas Gerais	Brazil
Virginia Kagure Wachira	Universidade de Brasília	Distrito Federal	Brazil
Vivian Castro Lemos	Universidade Estadual de Campinas	São Paulo	Brazil
Viviana Mariá Draeger	Universidade Federal de Santa Catarina	Santa Catarina	Brazil
Walter Ataalpa de Freitas Neto	Universidade Estadual de Feira de Santana	Bahia	Brazil
Wanderson de Oliveira	Hospital das Forças Armadas	Distrito Federal	Brazil
Waneska Alexandra Alves	Universidade Federal de Juiz de Fora	Minas Gerais	Brazil
Wanessa da Silva de Almeida	Fundação Oswaldo Cruz	Rio de Janeiro	Brazil
Werner de Andrade Muller	Universidade Federal de Pelotas	Rio Grande do Sul	Brazil
Wildo Navegantes de Araújo	Universidade de Brasília	Distrito Federal	Brazil
Wyllians Borelli	Universidade Federal do Rio Grande do Sul	Rio Grande do Sul	Brazil
Ximena Pamela Claudia Díaz Bermúdez	Universidade de Brasília	Distrito Federal	Brazil
Virgínia D. Carvalho	Universidade Federal de Alfenas	Minas Gerais	Brazil
Virginia Kagure Wachira	Universidade de Brasília	Distrito Federal	Brazil
Vivian Castro Lemos	Universidade Estadual de Campinas	São Paulo	Brazil
Viviana Mariá Draeger	Universidade Federal de Santa Catarina	Santa Catarina	Brazil
Walter Ataalpa de Freitas Neto	Universidade Estadual de Feira de Santana	Bahia	Brazil
Wanderson de Oliveira	Direção Técnica de Ensino e Pesquisa do Hospital das Forças Armadas	Distrito Federal	Brazil
Waneska Alexandra Alves	Universidade Federal de Juiz de Fora	Minas Gerais	Brazil
Wanessa da Silva de Almeida	Fundação Oswaldo Cruz	Rio de Janeiro	Brazil
Werner de Andrade Muller	Universidade Federal de Pelotas	Rio Grande do Sul	Brazil
Wildo Navegantes de Araújo	Universidade de Brasília	Distrito Federal	Brazil
Wyllians Borelli	Universidade Federal do Rio Grande do Sul	Rio Grande do Sul	Brazil
Ximena Pamela Claudia Díaz Bermúdez	Universidade de Brasília	Distrito Federal	Brazil

