# Supplementary material for: Appreciation message
Source: Epidemiol Serv Saude. 2025 Sep 8;34:e20240003. doi: 10.1590/S2237-96222025v34e20250003.en (PMC12447869; doi:10.1590/S2237-96222025v34e20250003.en)
Supplement: Translation

# Translation: Mensagem de agradecimento

**DOI:** 10.1590/S2237-96222025v34e20250003.pt

O Núcleo Editorial da *Epidemiologia e Serviços de Saúde: revista do SUS* (RESS) agradece aos revisores ad hoc abaixo listados pela colaboração e empenho na avaliação criteriosa dos manuscritos em 2024, o que contribuiu sobremaneira para a qualificação, a independência e o rigor ético do periódico.

**Table:** 

Nome	Instituição	Estado	País
Adail Afrânio Marcelino Nascimento	Secretaria Municipal da Saude de Fortaleza	Ceará	Brasil
Adeânio Almeida Lima	Faculdade Estácio Alagoinhas	Bahia	Brasil
Adeylson Guimarães Ribeiro	Universidade de São Paulo	São Paulo	Brasil
Adinaura Gama de Castro	Santa Casa de Misericórdia do Pará	Pará	Brasil
Adriana Barni Truccolo	Universidade Estadual do Rio Grande do Sul	Rio Grande do Sul	Brasil
Adriana Cezaretto	Universidade Nove de Julho	São Paulo	Brasil
Adriana Lúcia Meireles	Universidade Federal de Ouro Preto	Minas Gerais	Brasil
Adriana Sanudo	Universidade Federal de São Paulo	São Paulo	Brasil
Adriana Silva	Universidade Federal da Bahia	Bahia	Brasil
Adriana Tavares de Moraes Atty	Instituto Nacional de Câncer	Rio de Janeiro	Brasil
Andréa Cronemberger Rufino	Universidade Estadual do Piauí	Piauí	Brasil
Alberto Madeiro	Universidade Estadual do Piauí	Piauí	Brasil
Alcindo José Rosa	Universidade Federal do Mato Grosso	Mato Grosso	Brasil
Alex Sandro Rolland Souza	Instituto de Medicina Integral Professor Fernando Figueira	Recife	Brasil
Alexandre Tadashi Inomata Bruce	Secretaria Municipal de Saúde de Manaus	Amazonas	Brasil
Aline do Nascimento	Ministério da Saúde	Distrito Federal	Brasil
Aline Fernanda Silva Sampaio	Universidade Federal do Acre	Acre	Brasil
Aline Pilon	Ministério da Saúde	Distrito Federal	Brasil
Aline Pinto Marques	Fundação Oswaldo Cruz	Rio de Janeiro	Brasil
Aline Sales	Universidade Federal de Minas Gerais	Minas Gerais	Brasil
Aline Vieira de Lima	Ministério da Saúde	Distrito Federal	Brasil
Alisson Diego Machado	Universidade de São Paulo	São Paulo	Brasil
Almária Mariz Batista	Universidade Federal do Rio Grande do Norte	Rio Grande do Norte	Brasil
Aluísio de Oliveira	Instituto Federal do Sudeste de Minas Gerais	Minas Gerais	Brasil
Amanda Cilene Cruz Aguiar Castilho da Silva	Universidade Federal do Rio de Janeiro	Rio de Janeiro	Brasil
Amanda Coutinho de Souza	Organização Pan-Americana da Saúde	Distrito Federal	Brasil
Amanda Fonseca Medeiros	Universidade Federal de Minas Gerais	Minas Gerais	Brasil
Amanda Nathale Soares	Escola de Saúde Pública do Estado de Minas Gerais	Minas Gerais	Brasil
Ana Carolina Bezerra de Lima	Universidade de Pernambuco	Pernambuco	Brasil
Ana Carolina Feldenheimer da Silva	Universidade do Estado do Rio de Janeiro	Rio de Janeiro	Brasil
Ana Carolina Micheletti Gomide Nogueira de Sá	Universidade Federal de Minas Gerais	Minas Gerais	Brasil
Ana Coelho de Albuquerque	Instituto de Medicina Integral Prof. Fernando Figueira	Pernambuco	Brasil
Ana Débora Assis Moura	Secretaria da Saúde do Estado do Ceará	Ceará	Brasil
Ana Freitas Ribeiro	Instituto de Infectologia Emílio Ribas	São Paulo	Brasil
Ana Isabelle de Gois Queiroz	Centro Universitário Uniateneu	Ceará	Brasil
Ana Laura Brandão	Fundação Oswaldo Cruz	Rio de Janeiro	Brasil
Ana Lúcia Schaefer Ferreira de Mello	Universidade Federal de Santa Catarina	Santa Catarina	Brasil
Ana Lúcia Lyrio de Oliveira	Universidade de São Paulo	São Paulo	Brasil
Ana Maria Roselino	Universidade de São Paulo	São Paulo	Brasil
Ana Marli Christovam Sartori	Universidade de São Paulo	São Paulo	Brasil
Ana Patrícia Alves da Silva	Universidade de Fortaleza	Ceará	Brasil
Ana Paula da Costa Marques	Universidade Federal de Mato Grosso do Sul	Mato Grosso do Sul	Brasil
Ana Paula da Cunha	Fundação Oswaldo Cruz	Rio de Janeiro	Brasil
Ana Paula de Rocha Sales Miranda	Universidade Federal da Paraíba	Paraíba	Brasil
André Luis Alves de Quevedo	Secretaria Estadual de Saúde do Rio Grande do Sul	Rio Grande do Sul	Brasil
André Luiz Bigal	Universidade Federal de São Paulo	São Paulo	Brasil
André Luiz Machado das Neves	Universidade do Estado do Amazonas	Amazonas	Brasil
Andrea Moreira Arrué	Instituto Federal do Paraná	Paraná	Brasil
Andréa Tenório Correia da Silva	Universidade de São Paulo	São Paulo	Brasil
Andréia Insabralde de Queiroz-Cardoso	Universidade Federal de Mato Grosso do Sul	Mato Grosso do Sul	Brasil
Andrezza Santiago	Universidade Federal de Lavras	Minas Gerais	Brasil
Ângela Scalabrin	Santa Casa de São Paulo	São Paulo	Brasil
Angélica Atala Lombelo Campos	Universidade Federal de Juiz de Fora	Minas Gerais	Brasil
Angélica Ribeiro e Silva	Universidade Federal de Minas Gerais	Minas Gerais	Brasil
Angélica Yukari Takemoto	Centro Universitário Guairacá	Paraná	Brasil
Anke Bergmann	Instituto Nacional do Câncer	Rio de Janeiro	Brasil
Ann Jansen	Universidade Federal de Minas Gerais	Minas Gerais	Brasil
Antonio Augusto Ferreira Carioca	Universidade de Fortaleza	Ceará	Brasil
Antônio Carlos Frias	Universidade de São Paulo	São Paulo	Brasil
Antônio Carlos Vieira Ramos	Universidade do Estado de Minas Gerais	Minas Gerais	Brasil
Antonio da Silva Menezes Junior	Universidade Federal de Goiás	Goiás	Brasil
Antonio Gasparoto	Universidade Federal de Mato Grosso do Sul	Mato Grosso do Sul	Brasil
Antonio Ygor Modesto de Oliveira	Ministério da Saúde	Distrito Federal	Brasil
Ariela Mota Ferreira	Universidade Estadual de Montes Claros	Minas Gerais	Brasil
Ariene Carmo	Universidade Federal de Minas Gerais	Minas Gerais	Brasil
Arn Migowski Rocha dos Santo	Instituto Nacional do Câncer	Rio de Janeiro	Brasil
Arthur Duarte Fantesia Costa Cruz	Secretaria de Estado de Saúde de Mato Grosso do Sul	Mato Grosso do Sul	Brasil
Bárbara dos Santos Simões	Universidade Professor Edson Antônio Velano	Minas Gerais	Brasil
Betina Berlitz	Serviço Social do Comércio	Rio Grande do Sul	Brasil
Betine Pinto Moehlecke Iser	Universidade do Sul de Santa Catarina	Santa Catarina	Brasil
Breno de Oliveira Ferreira	Universidade Federal do Amazonas	Amazonas	Brasil
Brigina Kemp	Conselho de Secretários Municipais de Saúde do Estado de São Paulo	São Paulo	Brasil
Bruna Drumond Silveira	Fundação Oswaldo Cruz	Rio de Janeiro	Brasil
Bruna Moretti Luchesi	Universidade Federal de Mato Grosso do Sul	Mato Grosso do Sul	Brasil
Bruna Teles Soares Beserra	Universidade Federal de Minas Gerais	Minas Gerais	Brasil
Cacilda Tezelli Junqueira Padovani	Universidade Federal de Mato Grosso do Sul	Mato Grosso do Sul	Brasil
Caio Graco Zeppelini	Universidade Federal da Bahia	Bahia	Brasil
Caio Vieira de Barros Arato	Universidade Estadual de Campinas	São Paulo	Brasil
Carla Fabiana Tenanni	Faculdade de Medicina de Jundiaí	São Paulo	Brasil
Carla Rocha Pereira	Fundação Oswaldo Cruz	Rio de Janeiro	Brasil
Carla Luzia França Araújo	Universidade Federal do Rio de Janeiro	Rio de Janeiro	Brasil
Carlos Frederico S. Marques	Fundação Faculdade de Medicina	São Paulo	Brasil
Carlos José de Paula Silva	Universidade Federal de Minas Gerais	Minas Gerais	Brasil
Carmen Silvia Bruniera Domingues	Secretaria de Estado da Saúde de São Paulo	São Paulo	Brasil
Carolina Abreu Henn de Araújo	Universidade Federal de Santa Catarina	Santa Catarina	Brasil
Carolina de Campos Carvalho	Fundação Oswaldo Cruz	Rio de Janeiro	Brasil
Carolina Mariano Pompeo	Universidade Federal de Mato Grosso do Sul	Mato Grosso do Sul	Brasil
Carolina Muller Ferreira	Universidade Estadual de Campinas	São Paulo	Brasil
Caroline de Barros Gomes	Universidade Estadual Paulista	São Paulo	Brasil
Cássia Barbosa Reis	Universidade Estadual do Mato Grosso do Sul	Mato Grosso do Sul	Brasil
Cássia CP Mendicino	Secretaria de Estado de Saude de Minas Gerais	Minas Gerais	Brasil
Cassio Hartmann	Instituto Federal de Alagoas	Alagoas	Brasil
Cátia Regina Ficagna	Universidade Federal do Rio Grande do Sul	Rio Grande do Sul	Brasil
Catiane Costa Viana	Universidade Federal de Minas Gerais	Minas Gerais	Brasil
Christine Faustino	Universidade Estadual do Mato Grosso do Sul	Mato Grosso do Sul	Brasil
Cintia de Freitas Oliveira	Instituto de Saúde	São Paulo	Brasil
Clareci Silva Cardoso	Universidade Federal de São João del-Rei	Minas Gerais	Brasil
Cláudia Carolina Costa Braga	Universidade Estadual Paulista	São Paulo	Brasil
Claudia Cristina Lima de Castro	Secretaria de Saúde do Recife	Pernambuco	Brasil
Cláudia Raulino Tramontt	Universidade de São Paulo	São Paulo	Brasil
Claudia Tavares Regadas	Fundação Oswaldo Cruz	Rio de Janeiro	Brasil
Cláudia Volpe Chaves	Hospital Regional de Mato Grosso do Sul	Mato Grosso do Sul	Brasil
Claudio Gruber Mann	Universidade Federal do Rio de Janeiro	Rio de Janeiro	Brasil
Clineu de Mello Almada Filho	Universidade Federal de São Paulo	São Paulo	Brasil
Cristiane Helena Gallasch	Universidade do Estado do Rio de Janeiro	Rio de Janeiro	Brasil
Cristianny Miranda	Universidade Federal de Minas Gerais	Minas Gerais	Brasil
Cristiano Bertolossi Marta	Universidade do Estado do Rio de Janeiro	Rio de Janeiro	Brasil
Cristina Pimenta	Instituto Nacional de Infectologia Evandro Chagas(INI), Fiocruz	Rio de Janeiro	Brasil
Cristina Wide Pissetti	Universidade Federal da Paraíba	Paraíba	Brasil
Cristine Vieira do Bonfim	Fundação Joaquim Nabuco	Pernambuco	Brasil
Dalila de Carvalho Silva Gonzaga	Universidade de Brasília	Distrito Federal	Brasil
Daniel Hideki Bando	Universidade Federal de Alfenas	Minas Gerais	Brasil
Daniela de Almeida Pereira	Universidade Federal de Viçosa	Minas Gerais	Brasil
Danúbia Hillesheim	Universidade Federal de Santa Catarina	Santa Catarina	Brasil
Danyllo do Nascimento Silva Junior	Universidade Federal do Rio Grande do Norte	Rio Grande do Norte	Brasil
Dartagnan Pinto Guedes	Universidade Estadual do Norte do Paraná	Paraná	Brasil
David Coelho	Harvard University	Massachusets	Estados Unidos
Dayanne da Costa Maynard	Centro Universitário de Brasília	Distrito Federal	Brasil
Dayse Sanches Guimarães Paião	Universidade Federal da Grande Dourados	Mato Grosso do Sul	Brasil
Débora Castanheira	Fundação Oswaldo Cruz	Rio de Janeiro	Brasil
Débora Driemeyer Wilbert	Universidade Santo Amaro	São Paulo	Brasil
Denis Gonçalves Ferreira	Centro Universitário Várzea Grande	Mato Grosso	Brasil
Denise Eliziana de Souza	Escola Nacional de Saúde Pública Rio de Janeiro	Rio de Janeiro	Brasil
Denise Fortes	Secretaria de Estado de Saúde de Mato Grosso do Sul	Mato Grosso do Sul	Brasil
Deyvyd Manoel Condé Andrade	Secretaria Municipal de Saúde do Rio de Janeiro	Rio de Janeiro	Brasil
Diva Furtado Lacerda	Universidade Federal de Goiás	Goiás	Brasil
Divanise Suruagy Correia	Universidade Federal de Alagoas	Alagoas	Brasil
Doroteia Hofelmann	Universidade Federal do Paraná	Paraná	Brasil
Ed Wilson Rodrigues Vieira	Universidade Federal de Minas Gerais	Minas Gerais	Brasil
Edgar Oshiro	Escola de Saúde Pública Dr. Jorge David Nasser	Mato Grosso do Sul	Brasil
Eduardo Hage Carmo	Fundação Oswaldo Cruz	Distrito Federal	Brasil
Eduardo Jorge da Fonseca Lima	Instituto de Medicina Integral Professor Fernando Figueira	Pernambuco	Brasil
Elen Ferraz Teston	Universidade Federal de Mato Grosso do Sul	Mato Grosso do Sul	Brasil
Eliane Rolim de Holanda	Universidade Federal da Paraíba	Paraíba	Brasil
Eliete Albano de Azevedo Guimarães	Universidade Federal de São João del-Rei	Minas Gerais	Brasil
Elisa Prezotto Giordani	Universidade de São Paulo	São Paulo	Brasil
Elisabete Pereira Silva	Universidade Federal de Pernambuco	Pernambuco	Brasil
Elisabeth Meloni Vieira	Universidade de São Paulo	São Paulo	Brasil
Elisângela Aparecida da Silva Lizzi	Universidade Tecnológica Federal do Paraná	Paraná	Brasil
Elisete Duarte	Universidade Federal de Mato Grosso	Mato Grosso	Brasil
Eliseu Alves Waldman	Universidade de São Paulo	São Paulo	Brasil
Emile Danielly Amorim Pereira	Faculdade Edufor	Maranhão	Brasil
Emilia Moreira Jalil	Fundação Oswaldo Cruz	Rio de Janeiro	Brasil
Erick Soares Lisboa	Universidade Federal da Bahia	Bahia	Brasil
Erlandson Ferreira Saraiva	Universidade Federal de Mato Grosso do Sul	Mato Grosso do Sul	Brasil
Erly Catarina de Moura	Universidade de Brasília	Distrito Federal	Brasil
Ernani Tiaraju de Santa Helena	Universidade Regional de Blumenau	Santa Catarina	Brasil
Ester Souto	Fundação Oswaldo Cruz	Rio de Janeiro	Brasil
Eunice Francisca Martins	Universidade Federal de Minas Gerais	Minas Gerais	Brasil
Evelina Chapman	Fundação Oswaldo Cruz	Distrito Federal	Brasil
Expedito José de Albuquerque Luna	Universidade de São Paulo	São Paulo	Brasil
Fabiana Alves Soares	Universidade Federal do Maranhão	Maranhão	Brasil
Fabiana Baggio Nerbass	Fundação Pró-Rim	Santa Catarina	Brasil
Fabiana Schuelter-Trevisol	Universidade do Sul de Santa Catarina	Santa Catarina	Brasil
Fabiane Letícia de Freitas	Universidade de São Paulo	São Paulo	Brasil
Fabrizia Foletto	Universidade Federal de Mato Grosso do Sul	Mato Grosso do Sul	Brasil
Felipe Guimarães Tavares	Universidade Federal Fluminense	Rio de Janeiro	Brasil
Felipe Teixeira de Mello Freitas	Universidade do Distrito Federal	Distrito Federal	Brasil
Fernanda Esthefane Garrides Oliveira	Universidade Federal de Minas Gerais	Minas Gerais	Brasil
Fernanda Leticia Ferreira	Fundação Oswaldo Cruz	Rio de Janeiro	Brasil
Fernanda Marcelina Silva	Universidade de São Paulo	São Paulo	Brasil
Fernando Antônio Ribeiro de Gusmão-filho	Universidade de Pernambuco	Pernambuco	Brasil
Fernando Lopes Tavares de Lima	Instituto Nacional do Câncer	Rio de Janeiro	Brasil
Fernando Silva Guimarães	Universidade Federal de Pelotas	Rio Grande do Sul	Brasil
Flávia Casale Abe	Universidade de Sorocaba	São Paulo	Brasil
Flavia Aparecida Dias Marmo	Universidade Federal do Triangulo Mineiro	Minas Gerais	Brasil
Flávia Meneguetti Pieri	Universidade Estadual de Londrina	Paraná	Brasil
Flávia Mori Sarti	Universidade de São Paulo	São Paulo	Brasil
Flávia Reis de Andrade	Universidade de Brasília	Distrito Federal	Brasil
Flávio Renato Reis de Moura	Universidade La Salle	Rio Grande do Sul	Brasil
Franciele Costa da Silva Perez	Universidade Estadual Paulista	São Paulo	Brasil
Francilene Jane Pereira	Universidade Federal da Paraiba	Paraíba	Brasil
Francisco Inácio Pinkusfeld Bastos	Fundação Oswaldo Cruz	Rio de Janeiro	Brasil
Francisco Tustumi	Universidade de São Paulo	São Paulo	Brasil
Gabriel Pavinati	Universidade Estadual de Maringá	Paraná	Brasil
Gabriel Zorello Laporta	Centro Universitário da Faculdade de Medicina do ABC	São Paulo	Brasil
Gabriela Maria Cavalcanti Costa	Universidade Estadual da Paraíba	Paraíba	Brasil
Gabriela Soledad Márdero García	Universidade Federal do Ceara	Ceará	Brasil
Gabriella Morais Duarte Miranda	Universidade Federal de Pernambuco	Pernambuco	Brasil
Galba Freire Moita	Ministério da Saúde	Distrito Federal	Brasil
George Jó Bezerra Sousa	Ministério da Saúde	Distrito Federal	Brasil
Gerson Barbosa	Instituto Pasteur de São Paulo	São Paulo	Brasil
Gerson Luiz Marinho	Universidade Federal do Rio de Janeiro	Rio de Janeiro	Brasil
Giselle Freitas	Universidade Federal de Minas Gerais	Minas Gerais	Brasil
Giulia Silva	Universidade de São Paulo	São Paulo	Brasil
Glaucia Maria Bressan	Universidade Tecnológica Federal do Paraná	Paraná	Brasil
Gláucio de Oliveira Nangino	Hospital da Polícia Militar de Minas Gerais	Minas Gerais	Brasil
Guilherme Diogo Silva	Universidade de São Paulo	São Paulo	Brasil
Gustavo Magno Baldin Tiguman	Universidade Estadual de Campinas	São Paulo	Brasil
Gustavo Santa Roza Saggese	Universidade de São Paulo	São Paulo	Brasil
Haroldo da Silva Ferreira	Universidade Federal de Alagoas	Alagoas	Brasil
Heitor Andrade	Universidade de São Paulo	São Paulo	Brasil
Heitor Victor Veiga da Costa	Universidade Federal de Pernambuco	Pernambuco	Brasil
Helena Ribeiro	Universidade de São Paulo	São Paulo	Brasil
Helierson Gomes	Universidade Federal do Norte do Tocantins	Tocantins	Brasil
Iana Mundim de Oliveira	Universidade Federal de Goiás	Goiás	Brasil
Icaro José Santos Ribeiro	Universidade Estadual de Santa Cruz	Bahia	Brasil
Igor Gonçalves Ribeiro	Ministério da Saúde	Distrito Federal	Brasil
Inara Cunha	Escola de Saúde Pública Dr. Jorge David Nasser	Mato Grosso do Sul	Brasil
Indianara Maria de Barros Canuto	Secretaria de Saúde do Recife	Pernambuco	Brasil
Inês Rugani Ribeiro de Castro	Universidade do Estado do Rio de Janeiro	Rio de Janeiro	Brasil
Inês Dourado	Universidade Federal da Bahia	Bahia	Brasil
Isabela Thaís Machado de Jesus	Universidade Federal de São Carlos	São Paulo	Brasil
Isabele Barboza Moura	Fundação Oswaldo Cruz	Rio de Janeiro	Brasil
Isabella da Motta Passos	Universidade do Estado do Amazonas	Amazonas	Brasil
Isabella Vitral Pinto	Instituto René Rachou	Minas Gerais	Brasil
Isadora Caixeta da Silveira Ferreira	Universidade Federal de Uberlândia	Minas Gerais	Brasil
Isis Carvalho	Ministério da Saúde	Distrito Federal	Brasil
Italo Wesley Oliveira Aguiar	Universidade Federal do Ceará	Ceará	Brasil
Jack Roberto Silva Fhon	Universidade de São Paulo	São Paulo	Brasil
Jacqueline de Almeida Goncalves Sachett	Universidade do Estado do Amazonas	Amazonas	Brasil
Janaina Fonseca Almeida Souza	Universidade Federal de Minas Gerais	Minas Gerais	Brasil
Jaqueline Arboit	Universidade Federal de Santa Maria	Rio Grande do Sul	Brasil
Jaqueline Dario Capobiango	Universidade La Salle	Rio Grande do Sul	Brasil
Jaqueline Gomes de Jesus	Instituto Federal do Rio de Janeiro	Rio de Janeiro	Brasil
Jardel Corrêa de Oliveira	Prefeitura Municipal de Florianópolis	Santa Catarina	Brasil
Jardeliny Corrêa de Penha	Universidade Federal do Ceará	Ceará	Brasil
Jean Ezequiel Limongi	Universidade Federal de Uberlândia	Minas Gerais	Brasil
Jeferson Agnelli	Universidade da Sorocaba	São Paulo	Brasil
Jesem Douglas Yamall Orellana	Leônidas e Maria Deane Institute Fiocruz Amazonas	Amazonas	Brasil
Jéssica Muzy Rodrigues	Fundação Oswaldo Cruz	Rio de Janeiro	Brasil
Jessica Rasquim Araujo	Universidade Federal de Minas Gerais	Minas Gerais	Brasil
Jessye Melgarejo do Amaral Giordani	Universidade Federal de Santa Maria	Rio Grande do Sul	Brasil
João Alberto Martins Rodrigues	Universidade Federal do Paraná	Paraná	Brasil
João Carlos Alchieri	Universidade Federal do Rio Grande do Norte	Rio Grande do Norte	Brasil
João Paulo Cola	Secretaria de Estado da Saúde do Espírito Santo	Espírito Santo	Brasil
Johnatan Martins Sousa	Universidade Federal de Goiás	Goiás	Brasil
Jonas Eduardo Monteiro dos Santos	Instituto Nacional do Câncer	Rio de Janeiro	Brasil
Jonas Loiola Gonçalves	Universidade Estadual do Ceará	Ceará	Brasil
José Eduardo Gomes Arruda	Universidade Federal do Pará	Pará	Brasil
José Eleutério Junior	Universidade Federal do Ceará	Ceará	Brasil
José Fernando Salvador Carrillo	Universidade de Sorocoba	São Paulo	Brasil
José Geraldo Leite Ribeiro	Faculdade de Ciências Médicas de Minas Gerais	Minas Gerais	Brasil
José Rodolfo Lucena	Faculdade Medicina do Sertão	Pernambuco	Brasil
Josy Maria de Pinho da Silva	Organização Pan-Americana da Saúde	Distrito Federal	Brasil
Julia Gomes Freitas	Universidade de São Paulo	São Paulo	Brasil
Juliana Fernandes Kabad	Universidade Federal do Mato Grosso	Mato Grosso	Brasil
Juliana Gonçalves Reis	Fundação Oswaldo Cruz	Rio de Janeiro	Brasil
Juliana Lustosa Torres	Universidade Federal de Minas Gerais	Minas Gerais	Brasil
Juliana Mara Flores Bicalho	Universidade Federal de São João del-Rei	Minas Gerais	Brasil
Juliana Pavan Zuliani	Fundação Oswaldo Cruz	Rondônia	Brasil
Juliana Quero Reimão	Faculdade de Medicina de Jundiaí	São Paulo	Brasil
Juliana Vallim Jorgetto Santos	Universidade Federal de São Paulo	São Paulo	Brasil
Junir Antonio Lutinski	Universidade Comunitária da Região de Chapecó	Santa Catarina	Brasil
Kaciane Corrêa Mochizuke	Secretaria de Saúde de Porto Murtinho	Mato Grosso do Sul	Brasil
Karin Romano Posseger	Universidade Federal do Ceará	Ceará	Brasil
Karine Ferreira Barbosa	Secretaria de Estado de Saúde de Mato Grosso do Sul	Mato Grosso do Sul	Brasil
Kátia Cristina Barbosa Ferreira	Universidade de Pernambuco	Pernambuco	Brasil
Katiusse Rezende Alves	Universidade Federal de Viçosa	Minas Gerais	Brasil
Keitty Regina Cordeiro de Andrade	Universidade de Brasília	Distrito Federal	Brasil
Kelly Alves Magalhães	Universidade Federal de Minas Gerais	Minas Gerais	Brasil
Kelly Cristina do Nascimento	Universidade de Pernambuco	Pernambuco	Brasil
Kellyn Kessiene de Sousa Cavalcante	Universidade Federal do Ceará	Ceará	Brasil
Kérilin Stancine Santos Rocha	Universidade Federal do Espírito Santo	Espírito Santo	Brasil
Laio Magno	Universidade do Estado da Bahia	Bahia	Brasil
Larissa Fortunato Araujo	Universidade Federal do Ceara	Ceará	Brasil
Larissa Loures Mendes	Universidade Federal de Minas Gerais	Minas Gerais	Brasil
Larissa Zepka Baumgarten Rodrigues	Fundação de apoio à saúde de Curitiba	Paraná	Brasil
Laura Inês Maricato Otani	Universidade de Sorocoba	São Paulo	Brasil
Lauro Ferreira da Silva Pinto Neto	Santa Casa de Vitória	Espírito Santo	Brasil
Laylla Ribeiro Macedo	Universidade Federal do Rio de Janeiro	Rio de Janeiro	Brasil
Leandro Aparecido Souza	Universidade de Sorocoba	São Paulo	Brasil
Lenir Aparecida Chaves	Universidade Federal de Minas Gerais	Minas Gerais	Brasil
Leonardo Trench	Universidade de São Paulo	São Paulo	Brasil
Letícia Russo	Universidade Estadual de Maringá	Paraná	Brasil
Lia Borges de Mattos Custodio	Universidade Estadual Paulista	São Paulo	Brasil
Liandro da Cruz Lindner	Faculdade das Américas	São Paulo	Brasil
Lidia Bezerra Barbosa	Universidade Federal de Alagoas	Alagoas	Brasil
Lidiane da Silveira Gouvea Toledo	Fundação Oswaldo Cruz	Bahia	Brasil
Lidiane Sousa	Universidade Federal de Minas Gerais	Minas Gerais	Brasil
Lígia Amparo	Universidade Federal da Bahia	Bahia	Brasil
Lilian Cristina Rezende	Universidade Federal de Minas Gerais	Minas Gerais	Brasil
Lílian Fernandes Amarante	Fundação Oswaldo Cruz Ceará	Ceará	Brasil
Lissandra Santos Degner	Universidade Federral da Bahia	Bahia	Brasil
Lívia de Lima Moura	Secretaria Municipal de Saúde do Rio de Janeiro	Rio de Janeiro	Brasil
Luana da Silva Baptista Arpini	Secretaria Estadual de Saúde do Espírito Santo	Espírito Santo	Brasil
Luana Silva Rodrigues de Araujo	Hospital Israelita Albert Einstein	São Paulo	Brasil
Lucas Vinícius de Lima	Universidade Estadual de Maringá	Paraná	Brasil
Lucélia Colares	Universidade Federal do Rio de Janeiro	Rio de Janeiro	Brasil
Lucélia dos Santos Silva	Hospital Albert Einstein	São Paulo	Brasil
Luciana Bihain Hagemann de Malfussi	Universidade Federal de Santa Catarina	Santa Catarina	Brasil
Luciana Freire de Carvalho	Universidade Federal do Rio de Janeiro	Rio de janeiro	Brasil
Luciana Martins Rozman	Universidade São Judas Tadeu	São Paulo	Brasil
Lucieni Conterno	Universidade Estadual de Campinas	São Paulo	Brasil
Luis Phillipe Nagem Lopes	Universidade do Estado do Rio de Janeiro	Rio de Janeiro	Brasil
Luísa Silveira da Silva	Universidade Federal de Pelotas	Rio Grande do Sul	Brasil
Luiz Antonio Bastos Camacho	Fundação Oswaldo Cruz	Rio de janeiro	Brasil
Manoel Carlos Sampaio de Almeida Ribeiro	Universidade Presbiteriana Mackenzie	São Paulo	Brasil
Marcela de Abreu Moniz	Universidade Federal Fluminense	Rio de Janeiro	Brasil
Marcelo Coelho Goiato	Universidade Estadual Paulista Júlio de Mesquita Filho	São Paulo	Brasil
Márcia Cristina Dalla Costa	Universidade Estadual do Oeste do Paraná	Paraná	Brasil
Marco Aurélio Palazzi Sáfadi	Santa Casa de São Paulo	São Paulo	Brasil
Marcos Augusto Moraes Arcoverde	Universidade Estadual do Oeste do Paraná	Paraná	Brasil
Marcos Cláudio Signorelli	Universidade Federal do Paraná	Paraná	Brasil
Marcos Pereira Santos	Universidade Federal da Bahia	Bahia	Brasil
Maria Alves	Secretaria Municipal de Saude de Dois Irmãos do Buriti	Mato Grosso do Sul	Brasil
Maria Amélia Veras	Santa Casa de São Paulo	São Paulo	Brasil
Maria Angélica Tavares de Medeiros	Universidade Federal de São Paulo	São Paulo	Brasil
Maria Auxiliadora Martins	Universidade Federal de Minas Gerais	Minas Gerais	Brasil
Maria Beatriz Kneipp Dias	Instituto Nacional do Câncer	Rio de Janeiro	Brasil
Maria Cecília Ramos de Carvalho	Universidade Federal de Minas Gerais	Minas Gerais	Brasil
Maria Cristina Antunes Willemann	Conselho de Secretarias Municipais de Saúde de Santa Catarina	Santa Catarina	Brasil
Maria Cristina Mazzaia	Universidade Federal de São Paulo	São Paulo	Brasil
Maria Cynthia Braga	Instituto Aggeu Magalhães	Recife	Brasil
Maria do Carmo Fernandez Lourenço Haddad	Universidade Estadual de Londrina	Paraná	Brasil
Maria Laura Louzada	Universidade de São Paulo	São Paulo	Brasil
Maria Lúcia Zanetti	Universidade de São Paulo	São Paulo	Brasil
Maria Luiza Ferreira Imburana da Silva	Universidade Federal de Pernambuco	Pernambuco	Brasil
Maria Rita Donalísio	Universidade Estadual de Campinas	São Paulo	Brasil
Mariana Aparecida Brozoski	Universidade de São Paulo	São Paulo	Brasil
Mariana Gabriel	Universidade de São Paulo	São Paulo	Brasil
Mariana Lopes Galante	Universidade de São Paulo	São Paulo	Brasil
Mariana Souza Lopes	Universidade Federal da Paraíba	Paraíba	Brasil
Marilia Mastrocolla de Almeida Cardoso	Ministério da Saúde	Distrito Federal	Brasil
Mário Círio Nogueira	Universidade Federal de Juiz de Fora	Minas Gerais	Brasil
Mario Jorge Sobreira da Silva	Instituto Nacional do Câncer	Rio de Janeiro	Brasil
Maristela Honório Cayetano	Universidade de Mogi das Cruzes	São Paulo	Brasil
Marlene Azevedo Magalhães Monteiro	Universidade Federal de Minas Gerais	Minas Gerais	Brasil
Marta Heloisa Lopes	Universidade de São Paulo	São Paulo	Brasil
Maryana Carmello da Costa	Universidade de São Paulo	São Paulo	Brasil
Matheus Santos Melo	Universidade Federal de Sergipe	Sergipe	Brasil
Mayara Nascimento de Vasconcelos	Universidade Estadual do Ceará	Ceará	Brasil
Mayara Secco Torres da Silva	Fundação Oswaldo Cruz	Rio de Janeiro	Brasil
Melissa Luciana de Araújo	Universidade Federal de Minas Gerais	Minas Gerais	Brasil
Mercedes de Oliveira Neto	Universidade do Estado do Rio de Janeiro	Rio de janeiro	Brasil
Michele Fernanda Borges da Silva	Fundação Oswaldo Cruz	Rio de Janeiro	Brasil
Michelle Ramos	Fundação Oswaldo Cruz	Rio de Janeiro	Brasil
Milton Luiz Gorzoni	Santa Casa de São Paulo	São Paulo	Brasil
Naiara Sperandio	Universidade Federal do Rio de Janeiro	Rio de Janeiro	Brasil
Nair Tavares Milhem Ygnatios	Universidade Federal de Minas Gerais	Minas Gerais	Brasil
Natacha Kalline de Oliveira	Universidade de São Paulo	São Paulo	Brasil
Natacha Toral	Universidade de Brasília	Distrito Federal	Brasil
Natália Daroit	Universidade Federal do Rio Grande do Sul	Rio Grande do Sul	Brasil
Nathália Guimarães	Universidade Federal de Minas Gerais	Minas Gerais	Brasil
Nathália Luíza Ferreira	Universidade Federal de Lavras	Minas Gerais	Brasil
Nathan Aratani	Universidade Federal de Mato Grosso do Sul	Mato Grosso do Sul	Brasil
Neide Aparecida Boldrini	Universidade Federal do Espírito Santo	Espírito Santo	Brasil
Nicole Pischel	Universidade de São Paulo	São Paulo	Brasil
Norma Suely de Oliveira Farias	Instituto de Saúde	São Paulo	Brasil
Osmar de Oliveira Cardoso	Universidade Federal do Piauí	Piauí	Brasil
Oswaldo Villa Campos	Universidade de São Paulo	São Paulo	Brasil
Oziris Simões	Santa Casa de São Paulo	São Paulo	Brasil
Pablo Cardozo Rocon	Universidade Federal de Mato Grosso	Mato Grosso	Brasil
Pamela Alejandra Saavedra	Conselho Federal de Farmácia	Distrito Federal	Brasil
Pantelis Varvaki Rados	Universidade Federal do Rio Grande do Sul	Rio Grande do Sul	Brasil
Paola Barbosa Marchesini	Ministério da Saúde	Distrito Federal	Brasil
Patricia Jaime	Universidade de São Paulo	São Paulo	Brasil
Patrícia Maria Oliveira Machado	Universidade Federal de Santa Catarina	Santa Catarina	Brasil
Patrícia Pinheiro de Freitas	Universidade Federal dos Vales do Jequitinhonha e Mucuri	Minas Gerais	Brasil
Pauline Lorena Kale	Universidade Federal do Rio de Janeiro	Rio de Janeiro	Brasil
Paulo Carrara de Castro	Santa Casa de Misericórdia de São Paulo	São Paulo	Brasil
Paulo Henrique Faria Domingues	Universidade de Brasília	Distrito Federal	Brasil
Pedro Arthur Araújo	Ministério da Saúde	Distrito Federal	Brasil
Pedro Câncio Neto	Universidade de São Paulo	São Paulo	Brasil
Pedro Castro	Pontifícia Universidade Católica	Rio de Janeiro	Brasil
Pedro Henrique Brito da Silva	Secretaria Municipal de Saúde de Senador Canedo	Goiás	Brasil
Policardo Gonçalves da Silva	Universidade do Estado de Minas Gerais	Minas Gerais	Brasil
Priscila de Oliveira Luz	Universidade de São Paulo	São Paulo	Brasil
Rafael Aiello Bomfim	Universidade Federal de Mato Grosso do Sul	Mato Grosso do Sul	Brasil
Rafael Claro	Universidade Federal de Minas Gerais	Minas Gerais	Brasil
Rafaela Soares Rech	Universidade Federal de Ciências da Saúde de Porto Alegre	Rio Grande do Sul	Brasil
Raphael de Freitas Saldanha	Fundação Oswaldo Cruz	Rio de Janeiro	Brasil
Raquel Aparecida Ferreira	Instituto René Rachou	Minas Gerais	Brasil
Raquel de Deus Mendonça	Universidade Federal de Ouro Preto	Minas Gerais	Brasil
Raquel Silveira Bello Stucchi	Universidade Estadual de Campinas	São Paulo	Brasil
Rayane Cavalcante Pereira Batista	Universidade de Brasília	Distrito Federal	Brasil
Raysa Álvarez	Universidade de São Paulo	São Paulo	Brasil
Regina Marques	Prefeitura do Município de São Paulo	São Paulo	Brasil
Regina Tamaki	Universidade de São Paulo	São Paulo	Brasil
Renata Adrielle Lima Vieira	Universidade Federal da Paraíba	Paraíba	Brasil
Renata Cordeiro Domingues	Faculdade Pernambucana de Saúde	Pernambuco	Brasil
Renata Paz Leal Pereira	Universidade de São Paulo	São Paulo	Brasil
Renato Kfouri	Sociedade Brasileira de Imunizações	São Paulo	Brasil
Reyce Koga	Secretaria Municipal de Saúde de Manaus	Amazonas	Brasil
Ricardo Gadelha de Abreu	Ministério da Saúde	Distrito Federal	Brasil
Ricardo José de Paula Souza e Guimarães	Instituto Evandro Chagas	Pará	Brasil
Roberta Costa Jorge	Universidade do Estado do Rio de Janeiro	Rio de Janeiro	Brasil
Roberta Pereira Niquini	Instituto Federal do Rio de Janeiro	Rio de Janeiro	Brasil
Rodrigo Citton Padilha dos Reis	Ministério da Saúde	Distrito Federal	Brasil
Rodrigo Pinheiro de Toledo Vianna	Universidade Federal da Paraíba	Paraíba	Brasil
Rosa Maria Soares Madeira Domingues	Fundação Oswaldo Cruz	Rio de Janeiro	Brasil
Rosalia Garcia Neves	Secretaria Estadual de Saúde do Rio Grande do Sul	Rio Grande do Sul	Brasil
Rosana Richtmann	Instituto Emílio Ribas	São Paulo	Brasil
Samuel Benjoino Ferraz Aquino	Universidade Federal de Mato Grosso do Sul	Mato Grosso do Sul	Brasil
Samuel Lopes dos Santos	Universidade Federal do Piauí	Piauí	Brasil
Sandra Brignol	Universidade Federal Fluminense	Rio de Janeiro	Brasil
Sandra Maria do Valle Leone de Oliveira	Fundação Oswaldo Cruz	Mato Grosso do Sul	Brasil
Sarah Arangurem Karam	Universidade Católica de Pelotas	Rio Grande do Sul	Brasil
Sávio Marcelino Gomes	Universidade Federal da Paraíba	Paraíba	Brasil
Sheilla Siedler Tavares	Centro Universitário Facens	São Paulo	Brasil
Sheyla Maria Leite	Ministério da Saúde	Distrito Federal	Brasil
Sidiany Mendes Pimentel	Instituto Federal do Tocantins	Tocantins	Brasil
Silvia Helena Mendonça de Moraes	Fundação Oswaldo Cruz	Mato Grosso do Sul	Brasil
Silvio Barberato-Filho	Universidade de Sorocoba	São Paulo	Brasil
Sonia Silvestrin	Secretaria Municipal de Saúde de Porto Alegre	Rio Grande do Sul	Brasil
Soraya S Smaili	Universidade Federal de São Paulo	São Paulo	Brasil
Stela Lemos	Universidade Federal de Minas Gerais	Minas Gerais	Brasil
Tânia Cristina de Mattos Barros Petraglia	Centro de Referência para Imunobiológicos Especiais Myrtes Amorelli Gonzaga	Rio de janeiro	Brasil
Tania Maria de Araujo	Universidade Estadual de Feira de Santana	Bahia	Brasil
Tatiana Resende Prado Rangel Oliveira	Pontifícia Universidade Católica de Minas Gerais	Minas Gerais	Brasil
Tatiana Sadalla Collese	Centro Universitário São Camilo	São Paulo	Brasil
Tayanny Margarida Menezes Almeida Biase	Universidade Estadual de Campinas	São Paulo	Brasil
Teresa Cristina Vieira Segatto	Secretaria de Saúde do Distrito Federal	Distrito Federal	Brasil
Thais Gularte Della Vechia	Universidade Federal de Pelotas	Rio Grande do Sul	Brasil
Thais Machado	Universidade de São Paulo	São Paulo	Brasil
Thaynã Ramos Flores	Universidade Federal de Pelotas	Rio Grande do Sul	Brasil
Thayssa Maluff de Mello	Universidade Federal de Mato Grosso do Sul	Mato Grosso do Sul	Brasil
Thereza Maria Magalhães Moreira	Universidade Estadual do Ceará	Ceará	Brasil
Thiago Torres	Fundação Oswaldo Cruz	Rio de Janeiro	Brasil
Valéria de Campos Orsi	Universidade de Sorocoba	São Paulo	Brasil
Valéria Valim	Universidade Federal do Espírito Santo	Espírito Santo	Brasil
Vandilson Pinheiro Rodrigues	Universidade Federal do Maranhão	Maranhão	Brasil
Vanessa Alves Ferreira	Universidade Federal de Minas Gerais	Minas Gerais	Brasil
Vanessa Coelho de Aquino Benjoino Ferraz	Secretaria Municipal de Saúde de Campo Grande	Mato Grosso do Sul	Brasil
Vanessa Cruz Santos	Universidade Federal do Rio de Janeiro	Rio de Janeiro	Brasil
Vanessa Gomes Lima	Universidade Estadual de Campinas	São Paulo	Brasil
Vanessa Moraes Bezerra	Universidade Federal da Bahia	Bahia	Brasil
Vanessa Ramos Kirsten	Universidade Federal de Santa Maria	Rio Grande do Sul	Brasil
Vânia Paula Stolte-Rodrigues	Universidade Federal de Mato Grosso do Sul	Mato Grosso do Sul	Brasil
Vanner Boere Souza	Universidade Federal do Sul da Bahia	Bahia	Brasil
Vicente Sperb Antonello	Hospital Fêmina	Rio Grande do Sul	Brasil
Vinícius Gonçalves de Paula	Universidade Federal de Minas Gerais	Minas Gerais	Brasil
Virgina Weffort	Universidade Federal do Triangulo Mineiro	Minas Gerais	Brasil
Virgínia D. Carvalho	Universidade Federal de Alfenas	Minas Gerais	Brasil
Virginia Kagure Wachira	Universidade de Brasília	Distrito Federal	Brasil
Vivian Castro Lemos	Universidade Estadual de Campinas	São Paulo	Brasil
Viviana Mariá Draeger	Universidade Federal de Santa Catarina	Santa Catarina	Brasil
Walter Ataalpa de Freitas Neto	Universidade Estadual de Feira de Santana	Bahia	Brasil
Wanderson de Oliveira	Hospital das Forças Armadas	Distrito Federal	Brasil
Waneska Alexandra Alves	Universidade Federal de Juiz de Fora	Minas Gerais	Brasil
Wanessa da Silva de Almeida	Fundação Oswaldo Cruz	Rio de Janeiro	Brasil
Werner de Andrade Muller	Universidade Federal de Pelotas	Rio Grande do Sul	Brasil
Wildo Navegantes de Araújo	Universidade de Brasília	Distrito Federal	Brasil
Wyllians Borelli	Universidade Federal do Rio Grande do Sul	Rio Grande do Sul	Brasil
Ximena Pamela Claudia Díaz Bermúdez	Universidade de Brasília	Distrito Federal	Brasil
